# Growth, weight gain and BMI in virally suppressed children on antiretroviral therapy with specific reference to dolutegravir

**DOI:** 10.1186/s12887-023-04143-6

**Published:** 2023-07-04

**Authors:** Erik Belfrage, Sandra Soeria-Atmadja, Lars Navér

**Affiliations:** 1 Department of Pediatrics, Karolinska University Hospital, Huddinge 14186, K76-78 Stockholm, Sweden; 2 Division of Paediatrics, Department of Clinical Science, Intervention and Technology (CLINTEC), Karolinska Institutet, Stockholm, Sweden

**Keywords:** HIV, Antiretroviral, Dolutegravir, TAF, Children, Adolescents, Height, Weight, Growth, BMI, Metabolism, Patient care

## Abstract

**Background:**

Pediatric HIV infection cause retardation in height and weight. However, effective antiretroviral therapy (ART) result in desirable weight gain. Concerns have emerged regarding excessive weight gain related to the integrase inhibitor dolutegravir in adults but knowledge about the circumstances in children/adolescents is limited. We studied if dolutegravir containing ART or switch to dolutegravir affected body mass index (BMI) and described height development in the Stockholm pediatric/adolescent HIV cohort.

**Methods:**

A retrospective cohort study of height, weight and BMI in relation to ART in 94 children/adolescents living with HIV.

**Results:**

At last documented visit 60/94 children/adolescents were on dolutegravir, 50 had switched from a protease inhibitor or non-nucleoside reverse transcriptase inhibitor. Height standard deviation score (SDS) increased between first and last visit from mean height SDS -0.88 (16 had SDS < -2 and 6 SDS < -3) to -0.32 (four had SDS < -2). Mean BMI SDS increased from -0.15 to 0.62 in girls, but not (-0.20 to 0.09) in boys. The number of girls ≥ 12 years with BMI SDS ≥ 2 increased significantly from 0/38 to 8/38 and totally 9/50 (18%) girls and 4/44 (9%) boys had BMI SDS ≥ 2 at last visit. There was no difference in height or weight gain between different ART regimens. BMI SDS remained stable in 22/50 children switching to dolutegravir, decreased in 13 and increased in 15.

**Conclusion:**

Adolescent girls gained weight to a greater extent than expected but independently of ART. We found no association between dolutegravir alone or combined with tenofovir alafenamide fumarate (TAF) and excessive weight gain. Height development was within normal range.

## Introduction

In the absence of antiretroviral therapy (ART), HIV infection in children cause retardation in height and weight [1, 2]. Early diagnosis and effective treatment result in major improvement in previously growth retarded children [3, 4] and only children severely stunted before treatment initiation end up with final height growth retardation [5–8]. On the other hand, risk for increased weight gain during ART has been observed without conclusive evidence of a causative effect related to specific antiretroviral drugs. The mechanisms by which different antiretroviral agents would contribute to weight gain are unknown and the background to the observed weight gain seems to be multifactorial. Demographic factors, HIV status and composition of the ART regimen may contribute [9].

Specific concerns have been raised for excessive weight gain during treatment with integrase inhibitors (INSTI) and especially dolutegravir (DTG) [10–12]. However, data is conflicting and both absence of as well as increase in weight associated to DTG have been reported [3, 12]. Specific concerns have been raised about the combination of DTG and tenofovir alafenamide fumarate (TAF) [11]. Switch from TDF to TAF has been associated with weight gain irrespective of core antiretroviral agent [13]. Knowledge about children and adolescents, DTG, TAF and weight gain is limited. To date, a few published studies have reported an increase in body mass index (BMI) in children and adolescents living with HIV after switching to DTG-based ART [14–16]. No randomized controlled trial of excessive weight gain related to certain antiretrovirals (ARV) has been performed.

WHO recommend DTG as first line therapy in HIV infection in adults and children from six years of age in combination with two nucleoside reverse transcriptase inhibitors (NRTIs) [17]. INSTIs are generally well tolerated compared to protease inhibitors (PIs) and non-nucleoside reverse transcriptase inhibitors (NNRTIs) and has a high barrier towards development of antiretroviral resistance. Overweight and obesity is an emerging global problem, in low-, middle- and high-income countries [18, 19], which make interpretation of weight gain during certain treatment regimens difficult.

The aim of this study was to present demographic data and growth parameters for all children born 1996- 2015 from a single center cohort representing more than 50% of children living with HIV in Sweden and especially to study if switch to DTG affected BMI and to describe final adult height in the cohort.

## Methods

### Patients and setting

A retrospective cohort study. All children born 1996- 2015 nd followed at the pediatric HIV clinic at Karolinska University Hospital, Stockholm, Sweden were considered for inclusion. The children were routinely followed every three months with HIV RNA, CD4-cell count and routine lab tests, except some children who were partly followed at a home clinic, which regularly shared data with Karolinska. Height, weight and thus body mass index (BMI) were documented at each visit. Exclusion criteria was less than five registration points during the study period.

### Treatment recommendations

Effective pediatric ART has been available in Stockholm since 1996 and treatment recommendations from the Swedish Reference Group for Antiviral Therapy (RAV) have been updated on a regular basis [20, 21]. Until 2015 the recommended first line therapy for most age groups was a combination of a PI or Efavirenz (EFV) with two NRTIs. Thereafter, DTG was increasingly used in first line combinations.

### Documentation and sampling

A retrospective register-based cohort study. The data was collected August 2020 – December 2020 for those who were still patients at the clinic. For those who had been transferred to adult clinic, the end point was no later than 19 years of age.

Viral load, CD4-cell count and treatment history were retrieved from the Swedish national web-based quality registry InfCareHIV [22] and growth data from the electronic medical records (TakeCare).

Weight, height and BMI were converted to standard deviation scores (SDS) according to the WHO references [23, 24], and were used for comparison between different timepoints. BMI and BMI SDS calculated from WHO growth charts was recorded one year before switch to DTG, at switch and one year after switch. Variations in BMI less than ± 0.25 SDS were considered as no change, as BMI is sensitive to accuracy of height measurements. Height less than SDS < -2 was defined as stunted. According to the WHO references obesity is defined as BMI-SDS ≥ 2 [23, 24]. We added information about ISO-BMI for comparison to other studies. International Obesity Task Force (IOTF) (used in Sweden) defines overweight as 25 ≤ ISO-BMI < 30 and obesity as ISO-BMI ≥ 30 in children between 2- 18 years [25].

### Statistical analysis

Differences in distribution were tested with Fischer´s exact test, Pearson Chi-squared test and McNamar’s test. Two-sample t-test and dependent samples t-test were used for comparison of means. The statistical software JMP ver. 16.1.0d, SAS Institute Inc., Cary, North Carolina, USA was used.

## Results

### Characteristics of the study population

**Fig. 1:**
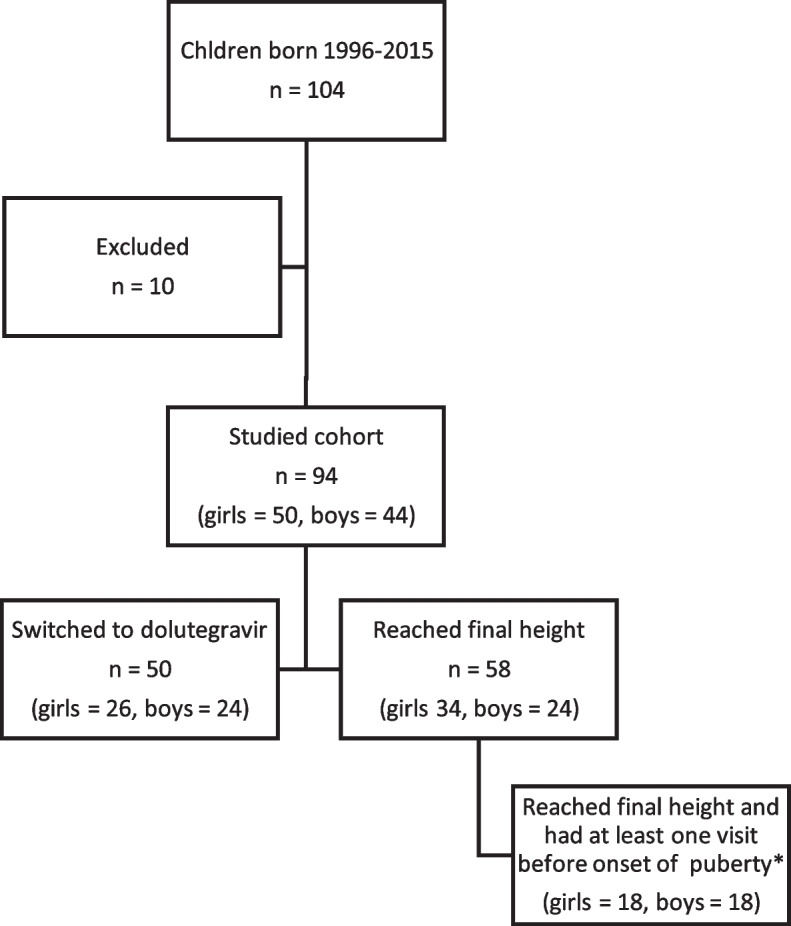
Cohort description. * Prepubertal age defined as < 9.5 years for girls and < 11.5 years for boys

Out of 104 children in the cohort born 1996– 2015, 94 children on ART with data from last follow up 2020 or after 18 years of age (50 girls, 44 boys) were included. Nine children were excluded because of less than five visits to the clinic and insufficient growth data the last year of follow up (Fig. 1). One untreated girl was excluded. Forty-four children were transferred to adult clinic and had data registered up to the age of 19.

The majority, 91 children, were born abroad and had immigrated with one or two caregivers (*n* = 60), moved to a relative living in Sweden (*n* = 7), arrived unaccompanied without caregiver (*n* = 4) or were internationally adopted (*n* = 23) (Table 1). No child was born in Sweden to a mother whose HIV status was known at pregnancy and delivery, but three inborn children were later found to be positive after the mother had tested positive (Table 1). These mothers had immigrated during late pregnancy and not attended the maternal health care program with routine HIV testing. Seventy-six of the children originated from Africa, 14 from Asia, two from the Americas and two from Europe. Mean age at arrival was 6 years and 2 months (Table 1).

**Table 1:** Characteristics of study participants

	**N**	**Age at arrival in Sweden** **mean (range)**	^**a**^**Continent of origin**	**Care at arrival**	**Age at ART start** **Mean (range)**	**Time on ART** **Mean (range)**	**Age at last visit** **mean (range)**	**Last CD4** **(× 10**^**6**^**)** **mean (range)**	**Last HIV RNA** **Percent < 50 copies/mL**
**All**	94	6y 2 m (0-16y 4 m)	76 Africa 14 Asia 2 Americas 2 Europe	23 int adopted 60 parent(s) 7 relative(s) 4 unaccompanied	6y 3 m (0y 2 m-17y 4 m)	8y 7 m (1y 2 m-17y 7 m)	14y 11 m (4y 10 m-19y 7 m)	766 (310–2120)	94%
**Girls all**	50	6y 8 m 0-16y 4 m)	38 Africa 10 Asia 2 Americas	12 int adopted 32 parent(s) 4 relatives 2 unaccompanied	6y 6 m (0y 4 m-16y 8 m)	8y 6 m (1y 2 m-17y 7 m)	15y 0 m (6y 5 m-19y 7 m)	816 (300–2120)	92%
**Girls < 12y**	12	3y 8 m (0-8y 8 m)	6 Africa 5 Asia 1 Americas	10 int adopted 2 parent(s)	1y 9 m (0y 4 m-4y 11 m)	6y 11 m (3y 3 m-10y 9 m)	8y 7 m (6y 5 m-11y 4 m)	1212 (550–2120)	100%
**Girls ≥ 12y**	38	7y 7 m (0-16y 4 m)	32 Africa 5 Asia 1 Americas	2 int adoption 30 parent(s) 4 relative(s) 2 unaccompanied	8y 0 m (0y 5 m-16y 8 m)	8y 9 m (1y 2 m-17y 7 m)	17y 0 m (12y 1 m-19y 7 m)	691 (300–1510)	90% (4 > 50 copies/mL: 86, 69, 109, 112)
**Boys all**	44	5y 7 m (0-16y 2 m)	38 Africa 4 Asia 2 Europe	11 int adopted 28 parent(s) 3 relative(s) 2 unaccompanied	6y 0 m (0y 2 m-17y 4 m)	8y 8 m (1y 5 m-17y 4 m)	14y 10 m (4y 10 m-18y 11 m)	709 (310–1320)	95%
**Boys < 12y**	12	3y 1 m (0-8y 1 m)	9 Africa 3 Asia	5 int adopted 6 parent(s) 1 relative(s)	3y 3 m (0y 2 m-7y 11 m)	5/10 (2y 0 m-12y 2 m)	9y 0 m (4y 10 m-11y 4 m)	873 (470–1320)	100%
**Boys ≥ 12y**	32	6y 6 m (0-16y 2 m)	29 Africa 1 Asia 2 Europe	6 int adopted 22 parent(s) 2 relative(s) 2 unaccompanied	7y 1 m (0y 3 m-17y 4 m)	9y 11 m (1y 5 m-17y 4 m)	17y 0 m (12y 5 m-18y 11 m)	648 (310–1050)	94% (2 > 50 c/mL: 73, 566)

*Y *Years, *m* Months, *SDS* Standard deviation score; int adopted = internationally adopted; parent(s) = arrived with one or two parents; relative(s) = arrived to relatives in Sweden; unaccompanied = arrived without caregiver;

^a^3 children born in Sweden were categorized to their mother’s continent of origin; *c* copies

### Treatment

ART was started before arrival in Sweden in 28 individuals (30%), within one year after arrival in 41 (44%). Treatment was deferred more than one year after diagnosis in Sweden in 25 (26%) children with high CD4-cell count, especially early in the study period before a treat all strategy was adapted. Timing of treatment initiation and choice of drug to start with did not differ between girls and boys. Fifty-three (56%) started with a PI as core agent, 31 (33%) with an NNRTI and 10 (11%) with DTG. As recommendations have changed, several children were successively switched to DTG and at last visit 60 (64%), 31 boys and 29 girls, were on DTG (Table 2). In total, fifty-eight children switched to a second line regimen. The reason for switch was in all but three cases treatment simplification and a potential of less side effects. The switches were executed during viral suppression and adequate CD4-cell counts. Two children were switched because of suspected side-effects related to their PI or NNRTI regimens and one reported headache after initiating DTG and switched successfully to a PI based regimen. The mean time on ART was 8 years and 7 months (Table 1). Regular yearly quality assessments of the Swedish quality register InfCareHIV showed repeated viral load < 50 copies/mL in > 92% of the cohort.

**Table 2:** Timing of initiation of ART and core agent at first and last visit respectively

		**Initiation of ART**			**First ART (core agent)**			**ART (core agent) at last visit**			**TAF at last visit**
	**N**	**Before arrival**	** < 1 year after arrival**	**Delayed ≥ 1 year**	**PI**	**NNRTI**	**DTG**	**PI**	**NNRTI**	**DTG**	**DTG + TAF**
**All**	94	28 (30%)	41 (44%)	25 (26%)	53 (56%)	31 (33%)	10 (11%)	17 (18%)	17 (18%)	60 (64%)	22 (23%)
** < 12 y**	24	13 (54%)	9 (38%)	2 (8%)	14 (58%)	8 (33%)	2 (8%)	3 (12%)	0 (0%)	21 (88%)	9 (38%)
** > ** **12 y**	70	15 (21%)	32 (46%)	23 (33%)	39 (56%)	23 (33%)	8 (11%)	14 (20%)	17 (24%)	39 (56%)	13 (19%)

*ART* Antiretroviral therapy, *PI* Protease inhibitor, *NNRTI* Non-nucleoside reverse transcriptase inhibitor, *DTG* Dolutegravir, *y* years

### Height at first and last visit and in relation to ART

Height SDS changed substantially between first and last visit. At first visit 16/94 (17%) had height SDS < -2 (Table 3) compared to 5/94 (4.2%) at last visit (*p* = 0.0004) and 5/94 (5.3%) had height SDS < -3 at first visit compared to none at last visit (*p* = 0.024.). The mean height SDS was -0.91(95% CI: -1.20- -0.62) at first visit and -0.33 (95% CI: -0.55- -0.12) at last (mean difference -0.58; *p* < 0.0001). The height SDS varied considerably between individuals and no difference between treatment regimens was observed.

**Table 3:** Height and BMI at first and last visit

**Gender**	**N**	**Stunted at first visit**	**Height SDS < -3 at first visit**	**Stunted at last visit**	^a^**BMI SDS ≥ 2** **at first visit**	^b^**BMI SDS ≥ 2 at last visit**	^b^**ISO-BMI ≥ 25 at first visit**	^b^**ISO-BMI ≥ 30 at first visit**	**25 ≤ **^b^**ISO-BMI < 30 at last visit**	^b^**ISO-BMI ≥ 30 at last visit**
**All**	94	16	5	5	3	13 (14%)	7	0	12 (13%)	7 (7%)
**Girls all**	50	6	1	1	0	9 (18%)	3	0	9 (18%)	5 (10%)
**Girls < 12y**	12	3	0	0	0	1 (8%)	1	0	3 (25%)	0
**Girls ≥ 12**	38	3	1	1	0	8 (21%)	2	0	6 (16%)	5 (13%)
**Boys all**	44	8	4	4	3	4 (9%)	4	0	3 (7%)	2 (5%)
**Boys < 12y**	12	4	3	1	1	2 (17%)	1	0	2 (17%)	0
**Boys ≥ 12y**	32	6	1	3	2	2 (6%)	3	0	1 (3%)	2 (6%)

Stunted = Height SDS < -2

^a^BMI ≥ 2 SDS = obese according to WHO definition

^b^ISO-BMI > 25 = overweight according to IOTF definition; ISO-BMI ≥ 30 = obese according

to IOTF definition

### BMI at first and last visit

BMI SDS increased from mean -0.17 (95% CI: -0.43- 0.09) to 0.29 (95% CI: 0.005- 0.58) between first and last visit (mean difference 0.47; *p* = 0.0023) in the whole study population. Mean BMI SDS increased significantly in girls from -0.15 to 0.62 (*p* = 0.0026) but not in boys (-0.20 to 0.09; n.s.). In subgroups, mean BMI SDS increased between first and last visit in girls ≥ 12 years and boys < 12 years (*p* = 0.006 and 0.028 respectively) but not in girls < 12 years nor in boys ≥ 12 years.

Considerable variation between individuals was observed. The proportion of individuals with ≥ 2 BMI SDS differed between first and last visit in the whole study population (*p* = 0.007) and in girls (*p* = 0.002) but not in boys (n.s). An increase in the number of individuals with BMI SDS ≥ 2 was obvious in older girls. Between first and last documented visit, the proportion of individuals with BMI SDS ≥ 2 increased from 0/38 to 8/38 (Table 3) among girls ≥ 12 years (*p* = 0.003). No difference in the proportion of individuals with BMI SDS ≥ 2 between first and last visit was seen in girls < 12 years; boys < 12 years or boys ≥ 12 years. At last documented visit a BMI SDS ≥ 2 was observed in 9/50 (18%) of all girls and in 4/44 (9%) of all boys (Table 3) with no significant difference between the sexes.

### BMI in relation to core ART agent

BMI SDS did not differ between ART regimens at last visit. In 34 individuals on ART without DTG the mean BMI SDS was 0.13 (95% CI; -0.32- 0.58) and in 60 on DTG 0.39 (95% CI; 0.00- 0.77) (n.s.). Out of 38 girls ≥ 12 years, 18 were on DTG at last visit. They had mean BMI SDS 0.74 (95% CI; -0.032- 1.52) compared to mean BMI SDS 0.49 (95% CI: -0.09- 1.07) in the 20 girls not on DTG (n.s.). No significant difference in BMI SDS was detected in boys ≥ 12 years on DTG (*n* = 21) compared to other ART combinations (*n* = 11) nor in boys or girls < 12 years. First antiretroviral core agent did not influence BMI SDS at last visit. Start with DTG, PI and NNRTI resulted in mean BMI SDS 0.13, 0.17 and 0.56 respectively at last visit (n.s.).

### BMI in relation to DTG combined with TAF or other NRTIs

Out of all 60 children and adolescents treated with DTG on last visit, 22 were also on TAF (13 girls and 9 boys) (Table 2). Mean BMI SDS in 22 children with DTG + TAF was 0.43 (95% CI; -0.09- 0.96) compared to 0.36 (95% CI; -0.17- 0.90) in 38 children on DTG + 2 other NRTIs (n.s). Among those with DTG and TAF, 4/22 (18%) had BMI SDS ≥ 2 compared to 6/38 (16%) of those with DTG and two other NRTIs (n.s.). Eight girls ≥ 12 years were on DTG + TAF at last visit. They had mean BMI SDS 0.36 (95% CI; -0.62- 1.35) compared to 1.04 (95% CI; -0.25- 2.34) in 10 girls ≥ 12 years on DTG + 2 other NRTIs (n.s.). No difference in BMI SDS was detected in boys ≥ 12 years on DTG + TAF (*n* = 5) compared to DTG + 2 other NRTIs (*n* = 16), nor in boys and girls < 12 years.

### Switch to dolutegravir and BMI

**Fig. 2:**
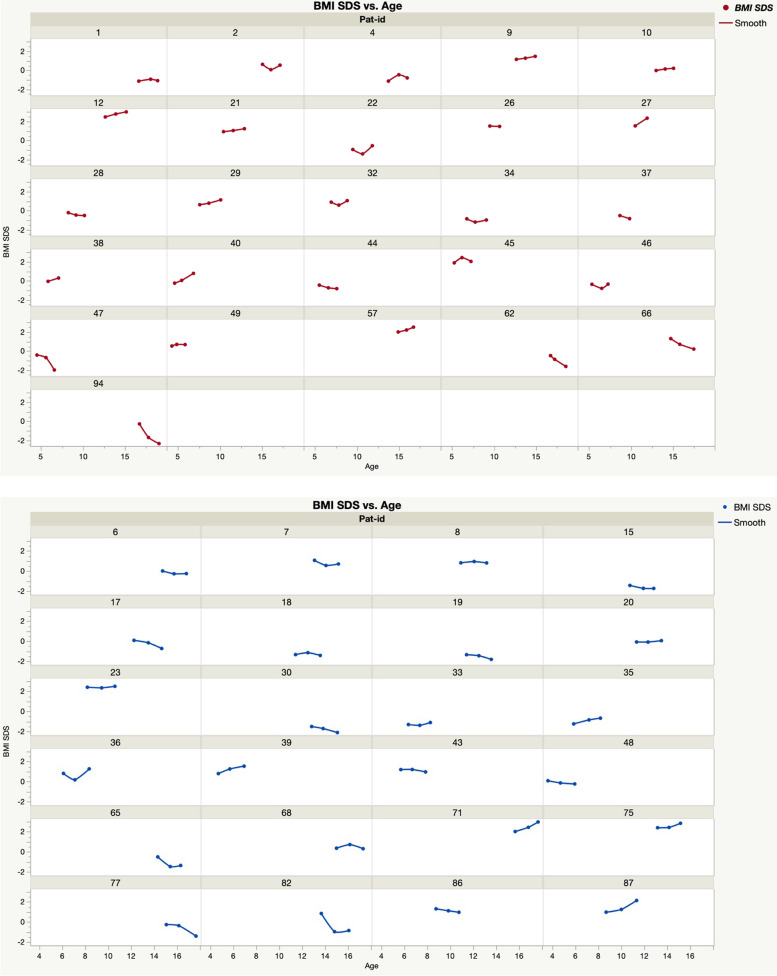
BMI SDS in individual children before, at and after switching to DTG. Girls = red; Boys = blue; Age (years); BMI = body mass index; SDS = standard deviation score

**Fig. 3:**
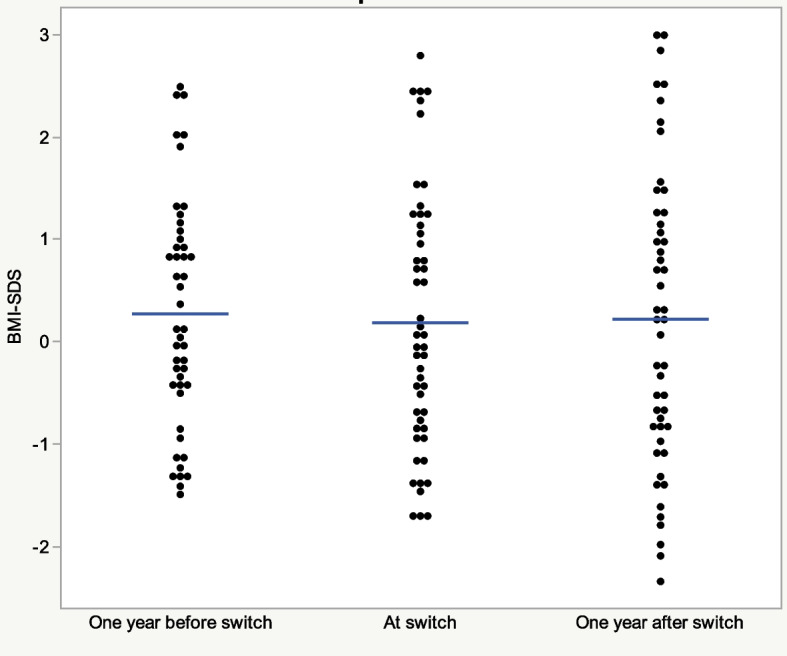
BMI SDS at different time points in relation to DTG switch. DTG = dolutegravir; BMI = body mass index; SDS = standard deviation score; blue line = mean; blue field = 95% CI

Ten children started on a DTG containing ART combination. Fifty switched to DTG (26 girls and 24 boys) from an NNRTI or PI based regimen while virally suppressed at a mean age of 11.26 years (95% CI: 10.11- 12.40). Individual BMI and BMI SDS data calculated one year before switch to DTG, at switch and one year after switch are presented in Fig. 2 and Table 4. No change in BMI SDS was seen one year after switch (*p* = 0.61; mean difference 0.035; 95% CI: -0.10- 0.17) (Fig. 3., Table 4). BMI SDS did not change in 22 children, decreased in 13 and increased in 15 (n.s) (Fig. 2, Table 5). However, individual BMI SDS curves indicate that an increase either started already before the DTG switch (*n* = 5) or appears after a previous decrease (*n* = 6) (Fig. 2, Table 5).

**Table 4:** BMI SDS related to DTG switch

		**N**	**Mean**	**Lower 95%**	**Upper 95%**	**SD**
**One year before DTG switch**	**Age (years)**	46	10.42	9.21	11.62	4.06
	**BMI SDS**	46	0.26	-0.07	0.60	1.12
**at DTG switch**	**Age (years)**	50	11.26	10.11	12.40	4.03
	**BMI SDS**	50	0.18	-0.18	0.53	1.25
**One year after DTG switch**	**Age (years)**	50	12.39	11.25	13.54	4.03
	**BMI SDS**	50	0.21	-0.20	0.62	1.46
**Delta BMI SDS**	**One year before DTG switch**	46	-0.12	-0.27	0.02	0.48
	**One year after DTG switch**	50	0.03	-0.10	0.17	0.48

BMI expressed as SDS relative to WHO reference; *BMI* Body mass index, *SDS* Standard deviation score, *SD* Standard deviation, *DTG* dolutegravir

**Table 5:** BMI SDS change in relation to DTG switch

**Group**		**N**	**Mean**	**Lower 95%**	**Upper 95%**	**SD**
**Decreased BMI SDS after switch to DTG**	**Age at switch**	13	13.15	10.72	15.59	4.03
	**BMI SDS at switch**	13	-0.40	-1.10	0.30	1.16
	**BMI SDS change after switch**	13	-0.56	-0.76	-0.38	0.31
**no change in BMI SDS after switch to DTG**	**Age at switch**	22	11.06	9.40	12.73	3.76
	**BMI SDS at switch**	22	0.22	-0.32	0.76	1.23
	**BMI SDS change after switch**	22	0.03	-0.03	0.09	0.14
**Increased BMI SDS after switch to DTG**	**Age at switch**	15	9.9	5.42	16.83	4.04
	**BMI SDS at switch**	15	0.61	-0.08	1.31	1.26
	**BMI SDS change after switch**	15	0.55	0.41	0.70	0.26

Evaluation of change in BMI after DTG switch based on change in BMI SDS, where changes within ± 0.25 SDS were considered as no change due to BMI being sensitive to accuracy of height measurements

*BMI* Body mass index, *SD* Standard deviation, *SDS* Standard deviation score, *DTG* Dolutegravir

### Growth from prepubertal age to adult age

**Fig. 4:**
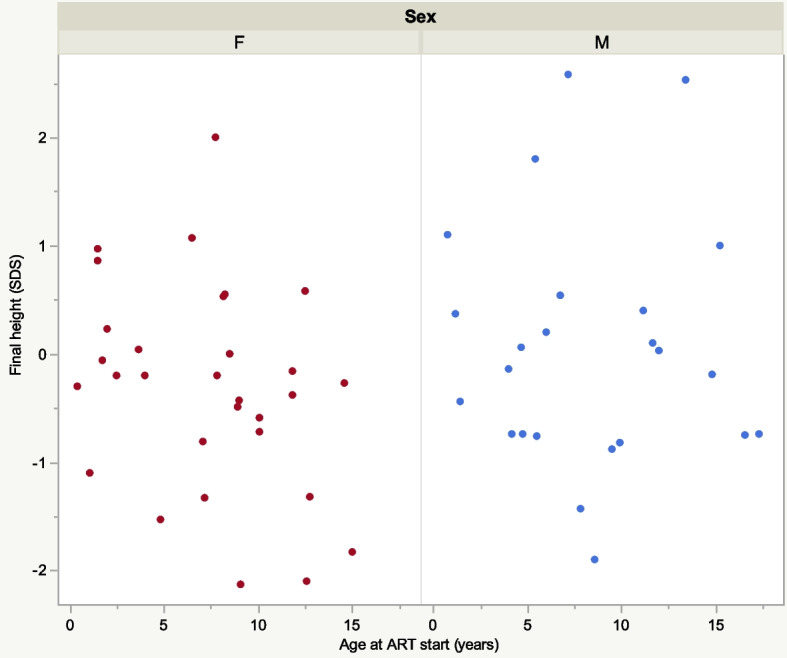
Age at ART start and adult height girls and boys. SDS = standard deviation score: ART = anti-retroviral therapy; F = female; M = male

Fifty-eight children reached final height (24 boys, 34 girls) where of 36 were followed since prepubertal age (18 boys, 18 girls) (Fig. 1). Height SDS and age at first visit, at start of puberty acceleration (growth velocity > 1 cm more than expected since last visit) and at last visit were recorded (Table 6). Interindividual variation was considerable (Fig. 4). Mean age at start of pubertal acceleration was 11.8 years in boys and 9.1 years in girls. Mean final height was 173.8 cm for boys and 160.6 cm for girls (Table 6) compared to the WHO reference 176 cm and 163 cm respectively.

**Table 6:** Growth in children with first visit during prepubertal ages and who reached final height

			**N**	**Mean**	**Lower 95%**	**Upper 95%**	**SD**
**Girls**	**First visit**	**Age (years)**	18	5.83	4.47	7.18	2.72
		**Height (cm)**	18	108.6	99.3	117.8	18.6
		**Height SDS**	18	-0.83	-1.30	-0.36	0.95
	**Pub acc**	**Age (years)**	18	9.14	8.42	9.86	1.45
		**Height (cm)**	18	129.4	125.3	129.4	8.15
	**Last visit**	**Age (years)**	18	17.26	16.61	17.91	1.30
		**Height (cm)**	18	160.6	157.4	163.8	6.36
		**Height SDS** ^a^	18	-0.26	-0.70	0.18	0.88
	**ART start**	**Age (years)**	18	6.30	4.35	8.26	3.94
**Boys**	**First visit**	**Age (years)**	18	5.45	4.03	6.87	2.85
		**Height (cm)**	18	108.4	98.5	118.3	19.9
		**Height SDS** ^a^	18	-0.55	-1.57	0.46	2.05
	**Pub acc**	**Age (years)**	18	11.81	11.27	12.34	1.08
		**Height (cm)**	18	147.2	133.0	169.5	10.1
	**Last visit**	**Age (years)**	18	17.82	17.38	18.29	0.91
		**Height (cm)**	18	173.8	169.8	177.9	8.14
		**Height SDS** ^a^	18	-0.25	-0.81	0.31	1.12
	**ART start**	**Age (years)**	18	6.23	4.51	7.95	3.46

Prepubertal age defined as < 9.5 years for girls and < 11.5 years for boys

*Pub acc* Pubertal acceleration, *SD* Standard deviation, *SDS* Standard deviation score, *ART* Antiretroviral therapy

^a^Calculated from age-specific reference values

## Discussion

We present a study about weight, height and BMI from a cohort of children and adolescents living with HIV, representing half of them living with HIV in Sweden, a high-income country with available resources for optimal pediatric HIV care. The cohort is representative for children/adolescents living with HIV in Sweden but differs from the general population and from cohorts of other chronic diseases since almost all are immigrants, mainly from Africa. In addition, only 2/3 lived with a biological parent and one fourth were internationally adopted (Table 1). The general adherence to ART was good and the degree of viral suppression was high throughout the study period.

The children recovered height after arrival in Sweden. The background to this is probably multi factorial and might be attributed to HIV diagnosis and treatment for those previously undiagnosed, effective treatment and follow-up for those previously not well treated in addition to better nutritional supply. A similar pattern has been observed in HIV negative internationally adopted children. The growth spurt occurred within the normal age span compared to the WHO growth charts and final height was within the normal range apart from a few children who were stunted already at arrival before ART initiation. This is similar to patterns described in HIV negative internationally adopted children [26, 27]. Reduced height in HIV-infected children was reported in studies from the pre-ART era [1, 2]. Final height in our cohort was almost within normal range compared to the WHO growth charts. This is similar to what was found in the large EPPICC cohort, where a recovery was seen after ART initiation, at least for those not severely stunted [8, 28] and children with height SDS ≥ -1 at ART initiation gained height similarly to HIV negative children at comparable age [8]. In the pre-ART era, a study of HIV-infected hemophilic boys in the UK showed normal growth the first 4.5 years after seroconversion [29], indicating that normal growth is expected if the child is not malnourished or severely affected by the HIV infection. The short final height in a few children in our cohort could be explained by factors like severe disease, malnutrition before immigration or genetic factors. Without knowledge of parental height, no certain interpretation is possible.

At last documented visit, 14 out of 50 (28%) of the girls and 5 out of 44 (12%) of the boys were overweight or obese defined as ISO-BMI ≥ 25. Thiscould be related to overweight and obesity in 14- 21% among girls and 10- 25% of boys aged 4- 18 years in the general Swedish adolescent population [30, 31]. A recent Swedish study shows that overweight is common already from 4 years of age (14- 18% in girls and 10–13% in boys) and increase to the numbers reported above in adolescents 13–19 years [31]. The referred studies used IOTF BMI cut-offs for overweight and obesity [25]. Boys were initially less prone to overweight than girls, but the opposite condition was seen among adolescents [30, 31]. In our study, obesity defined as BMI SDS ≥ 2 was observed in 9 out of 50 (18%) of all girls and in 4 out of 44 (9%) of all boys at last visit, while obesity defined according to IOTF (ISO-BMI ≥ 30) was observed in 5 out of 50 (10%) of the girls and 2 out of 44 (5%) of the boys (Table 3), which could be compared to 3- 4% (girls) and 2.7- 6% (boys) in the general population [30]. Thus, the degree of overweight and obesity may be higher among girls in our study than in the general population but as expected in boys [30, 31]. However, with a small cohort, the estimated proportions of overweight and obesity are uncertain making comparisons with.

There is concern in adults about possible excessive weight gain related to INSTIs and particularly DTG [11]. Especially women and none-whites have been reported to be at risk [10, 11]. However, a recent study suggested the difference in weight gain between DTG and EFV based regimens to be mainly related to impaired weight gain in slow metabolisers of EFV, due to possible side effects. Quick EFV metabolisers gained weight at similar rates as patients on DTG [32].

Little is published about a possible relation between DTG and weight gain in children and adolescents, but recently two studies have addressed excessive weight gain in relation to DTG switch [14, 15]. Transition to DTG in in 460 virally suppressed adolescents in Swaziland was associated with an increase in BMI change. Female adolescents experienced a larger change than males [15]. Notably, the conclusions were made without correction for puberty. In addition, boys and girls were analysed together in a cohort > 10 years of age, when age, puberty and sex are important parameters in analyses of BMI changes in children and adolescents. A smaller retrospective study of 38 children and youth (aged 0- 19 years) in the United States reported an increase in BMI change after switch to DTG. The median follow-up time was 527 days [14]. Neither did this study take puberty into account. In addition, the Odyssey study, not designed to study outcome of growth, compared DTG based therapy to standard of care in children and adolescents starting first- or second-line ART. BMI–for–age z score increased slightly more in the DTG group than in the standard of care group, but from low baseline levels. The weight gain occurred early and alongside a small increase in height, which was interpretated as improvement in normal growth [16].

We found no difference in weight gain between children and adolescents treated with DTG compared to other regimens. Among 50 children who switched to DTG, 22 kept their BMI position, compared to 13 and 15 with decrease or increase in BMI SDS respectively, suggesting absence of systematic increase in BMI after DTG switch. No change in BMI SDS could be seen 13 months after switch to DTG regardless of age and sex. We considered an observational time of 13 months as optimal to evaluate if a drug per se constitutes a risk factor for excessive weight gain. With longer observational time, other factors like environmental factors and changes in lifestyle will complicate the analysis.

Despite an increased number of adolescent girls with BMI SDS ≥ 2 between first (0/38) and last visit (8/38), there was no difference related to ART regimen. Eighteen individuals were on DTG at last visit and their BMI SDS did not differ from the 20 with an NNRTI or PI as core agent. Weight gain seen in HIV-infected individuals starting effective ART might be a normalization or adaption of BMI to levels seen in the general population rather than an excessive BMI increase caused by the different drugs. However, case reports of children and adolescents with excessive weight gain after shift to DTG [33] and reversion after change back to the initial ART combination might indicate the occurrence of certain individual factors predisposing for DTG related effects on weight. RCTs comparing the relationship between certain antiretrovirals and weight gain to healthy controls with similar socioeconomic background are lacking.

One strength of our study is the well-controlled, thoroughly monitored study cohort consisting of a considerable part of all available children and adolescents living with HIV in Sweden and another is taking puberty in account. To our knowledge it is the first study analysing DTG-related weight and BMI change to age adjusted growth charts and to the condition in the general population. We consider it a strength to have analyses of age adjusted change in BMI SDS with observations one year before, at switch and one year after switch to DTG. As a complement we also present the occurrence of overweight and obesity. Several studies define cut-off for suspected DTG-related weight gain as more than 7% increase in BMI from pre-ART BMI [11], which could represent unwanted weight gain but also reflect a desirable increase due to positive treatment effects. In addition, the 7% BMI increase does not take puberty in account. Thorough follow-up and repeated height and weight measurements guarantee good data quality. Despite representing half of the pediatric HIV population in Sweden, the cohort is small which limits definite conclusions regarding DTG and weight gain. Another limitation is the retrospective design.

In conclusion DTG was the dominating core agent at the last documented visit in this well treated pediatric cohort and we found no evidence of DTG and/or TAF causing excessive weight gain. The children and adolescents gained height and weight significantly between their first and last visit which might be an expected return to health effect caused by efficient ART. For those followed from prepubertal age, timing of pubertal growth and final height was within the normal range compared to the WHO growth standards. Short adult stature was seen only in those severely stunted before arrival in Sweden. Overweight and obesity in children and adolescents are growing problems in the general population as well as in this cohort. Adolescent girls gained weight to a greater extent than expected regardless of ART regimen but children/adolescents living with HIV and on effective ART essentially had a weight and height development like their Swedish peers.

## Data Availability

The datasets generated during and/or analysed during the current study are not publicly available due to data protection regulations but are available from the corresponding author on reasonable request.

## References

[1] Shannon KM, Ammann AJ (1985). Acquired immune deficiency syndrome in childhood. J Pediatr.

[2] Arpadi SM (2000). Growth failure in children with HIV infection. J Acquir Immune Defic Syndr.

[3] Jesson J, Koumakpai S, Diagne NR, Amorissani-Folquet M, Koueta F, Aka A (2015). Effect of Age at Antiretroviral Therapy Initiation on Catch-up Growth Within the First 24 Months Among HIV-infected Children in the IeDEA West African Pediatric Cohort. Pediatr Infect Dis J.

[4] McGrath CJ, Diener L, Richardson BA, Peacock-Chambers E, John-Stewart GC (2015). Growth reconstitution following antiretroviral therapy and nutritional supplementation: systematic review and meta-analysis. AIDS.

[5] Williams PL, Jesson J (2018). Growth and pubertal development in HIV-infected adolescents. Curr Opin HIV AIDS.

[6] Schomaker M, Leroy V, Wolfs T, Technau KG, Renner L, Judd A (2017). Optimal timing of antiretroviral treatment initiation in HIV-positive children and adolescents: a multiregional analysis from Southern Africa, West Africa and Europe. Int J Epidemiol.

[7] Bellavia A, Williams PL, DiMeglio LA, Hazra R, Abzug MJ, Patel K (2017). Delay in sexual maturation in perinatally HIV-infected youths is mediated by poor growth. AIDS.

[8] European Pregnancy and Paediatric HIV Cohort Collaboration study group (2019). Height and timing of growth spurt during puberty in young people living with vertically acquired HIV in Europe and Thailand. AIDS.

[9] Sax PE, Erlandson KM, Lake JE, McComsey GA, Orkin C, Esser S (2020). Weight Gain Following Initiation of Antiretroviral Therapy: Risk Factors in Randomized Comparative Clinical Trials. Clin Infect Dis.

[10] Eckard AR, McComsey GA (2020). Weight gain and integrase inhibitors. Curr Opin Infect Dis.

[11] Bansi-Matharu L, Phillips A, Oprea C, Grabmeier-Pfistershammer K, Gunthard HF, De Wit S (2021). Contemporary antiretrovirals and body-mass index: a prospective study of the RESPOND cohort consortium. Lancet HIV.

[12] Bourgi K, Rebeiro PF, Turner M, Castilho JL, Hulgan T, Raffanti SP (2020). Greater Weight Gain in Treatment-naive Persons Starting Dolutegravir-based Antiretroviral Therapy. Clin Infect Dis.

[13] Mallon PW, Brunet L, Hsu RK, Fusco JS, Mounzer KC, Prajapati G (2021). Weight gain before and after switch from TDF to TAF in a U.S. cohort study. J Int AIDS Soc..

[14] Koay WLA, Dirajlal-Fargo S, Levy ME, Kulie P, Monroe A, Castel AD (2021). Integrase Strand Transfer Inhibitors and Weight Gain in Children and Youth With Perinatal Human Immunodeficiency Virus in the DC Cohort. Open Forum Infect Dis..

[15] Thivalapill N, Simelane T, Mthethwa N, Dlamini S, Lukhele B, Okello V (2021). Transition to Dolutegravir is associated with an increase in the rate of body mass index change in a cohort of virally suppressed adolescents. Clin Infect Dis.

[16] Turkova A, White E, Mujuru HA, Kekitiinwa AR, Kityo CM, Violari A (2021). Dolutegravir as First- or Second-Line Treatment for HIV-1 Infection in Children. N Engl J Med.

[17] World Health Organization (2019). Update of recommendations on first- and second-line antiretroviral regimens.

[18] Collaboration NCDRF (2017). Worldwide trends in body-mass index, underweight, overweight, and obesity from 1975 to 2016: a pooled analysis of 2416 population-based measurement studies in 128.9 million children, adolescents, and adults. Lancet.

[19] Wijnhoven TM, van Raaij JM, Spinelli A, Rito AI, Hovengen R, Kunesova M (2013). WHO European Childhood Obesity Surveillance Initiative 2008: weight, height and body mass index in 6–9-year-old children. Pediatr Obes.

[20] Eriksen J, Carlander C, Albert J, Flamholc L, Gisslen M, Naver L (2020). Antiretroviral treatment for HIV infection: Swedish recommendations 2019. Infect Dis (Lond).

[21] Gisslen M, Ahlqvist-Rastad J, Albert J, Blaxhult A, Hamberg AK, Lindback S (2006). Antiretroviral treatment of HIV infection: Swedish recommendations 2005. Scand J Infect Dis.

[22] Andersson E, Nordquist A, Esbjornsson J, Flamholc L, Gisslen M, Hejdeman B (2018). Increase in transmitted drug resistance in migrants from sub-Saharan Africa diagnosed with HIV-1 in Sweden. AIDS.

[23] Group WHOMGRS (2006). WHO Child Growth Standards based on length/height, weight and age. Acta Paediatr Suppl.

[24] de Onis M, Onyango AW, Borghi E, Siyam A, Nishida C, Siekmann J (2007). Development of a WHO growth reference for school-aged children and adolescents. Bull World Health Organ.

[25] Cole TJ, Lobstein T (2012). Extended international (IOTF) body mass index cut-offs for thinness, overweight and obesity. Pediatr Obes.

[26] Stagi S, Papacciuoli V, Boiro D, Maggioli C, Ndambao NN, Losi S (2020). Auxological and endocrinological features in internationally adopted children. Ital J Pediatr.

[27] Proos LA (2009). Growth & development of Indian children adopted in Sweden. Indian J Med Res.

[28] Jesson J, Crichton S, Quartagno M, Yotebieng M, Collaborative Initiative for Paediatric HIVE, Research Global Cohort C (2022). Growth and CD4 patterns of adolescents living with perinatally acquired HIV worldwide, a CIPHER cohort collaboration analysis. J Int AIDS Soc.

[29] Pasi KJ, Collins MA, Ewer AK, Hill FG (1990). Growth in haemophilic boys after HIV infection. Arch Dis Child.

[30] Bygdell M, Celind J, Lilja L, Martikainen J, Simonson L, Sjogren L (2021). Prevalence of overweight and obesity from 5 to 19 years of age in Gothenburg. Sweden Acta Paediatr.

[31] Eriksson M, Lingfors H, Golsater M (2018). Trends in prevalence of thinness, overweight and obesity among Swedish children and adolescents between 2004 and 2015. Acta Paediatr.

[32] Griesel R, Maartens G, Chirehwa M, Sokhela S, Akpomiemie G, Moorhouse M (2021). CYP2B6 Genotype and Weight Gain Differences Between Dolutegravir and Efavirenz. Clin Infect Dis.

[33] Li J, Yusuf EH, Agwu AL (2021). Excessive Weight Gain Associated With Dolutegravir Initiation in a 10-Year-Old Female With Perinatally Acquired Human Immunodeficiency Virus: A Case Report and Review of the Literature. J Pediatric Infect Dis Soc.

